# Supplementary material for: CyTOF analysis identifies unusual immune cells in urine of BCG-treated bladder cancer patients
Source: Front Immunol. 2022 Sep 15;13:970931. doi: 10.3389/fimmu.2022.970931 (PMC9520259; doi:10.3389/fimmu.2022.970931)
Supplement: Supplementary file 1 [file DataSheet_1.pdf]

Supplementary Table 1. Cell count in urine

Patient	Week	Days after instillation	$x10^3$ cells/ml
P25	Before first instillation		9
	w1	d2	64
		d5	90
		d7	33
	w2	d7	95
	w3	d7	75
	w5	d7	1200
	w6	d5	720
		d7	2619
	w19	d2	75
		d5	53
		d7	22
P26	Before first instillation		40
	w1	d5	159
		d7	94
	w2	d7	80
	w3	d2	100
		d7	30
	w6	d5	18
		d7	9
	w7	d2	64
	w18	d7	4
P27	Before first instillation		0
	w2	d7	10
	w5	d7	43
P28	Before first instillation		0
	w2	d7	60
	w5	d7	188
P29	Before first instillation		0
	w2	d7	9
P30	Before first instillation		0
	w2	d7	10
	w3	d2	248
		d7	94
	w6	d2	200
		d7	9
P31	Before first instillation		4800
	w1	d2	1660
		d7	1510
	w2	d7	720
	w3	d2	1089
		d7	750
P41	w5	d7	4
P42	w2	d7	12
P44	w5	d7	12
P45	w5	d7	12
P46	w2	d7	12
P47	w5	d7	60

Supplementary Table 2. CyTOF antibody panel

Isotope	Ab	Clone	Company
Qdot - Cd	HLA-DR	Tu36	Life technologies
89Y	EpCAM	9C4	Biolegend
115In	CD19	HIB19	Biolegend
141Pr	CD38	HIT2	BioLegend
142Nd	CD11b	ICRF44	Biolegend
143Nd	CD4	OKT4	Biolegend
144Nd	CD8	SK1	Biolegend
145Nd	CD57	HCD57	Biolegend
146Nd	PD-1	EH12.2H7	Biolegend
147Sm	CD3	UCHT1	Biolegend
148Nd	CD35	E11	Biolegend
149Sm	TLR2	TL2.1	Biolegend
150Nd	CD45	HI30	Biolegend
151Eu	CD15	HI98	Biolegend
152Sm	CD63	H5C6	Biolegend
153Eu	CD11c	Bu15	Biolegend
154Sm	CD14	M5E2	Biolegend
155Gd	NKp46	9E2	Biolegend
156Gd	TCRgd	B1	Biolegend
157Gd	NKG2C	134522	R&D System
158Gd	CD24	ML5	Biolegend
159Tb	CD7	CD7-6B7	Biolegend
160Gd	CD69	FN50	Biolegend
161Dy	CD66b	G10F5	Biolegend
162Dy	CD64 (FcgrI)	10,1	Biolegend
163Dy	Invariant NKT	6B11	Biolegend
164Dy	CD89	A59	Biolegend
165Ho	CD107a	H4A3	Biolegend
166Er	TLR4	HTA125	Biolegend
167Er	CD94	DX22	Biolegend
168Er	MICA	MAB13001	R&D System
169Tm	CD32 (FcgrIIa)	FUN-2	Biolegend
170Yb	CD107b	H4B4	Biolegend
171Yb	NKG2A	131411	R&D systems
172Yb	CD25	M-A251	Biolegend
173Yb	CD95	DX2	Biolegend
174Yb	CD56	NCAM16.2	BD
175Lu	CD33	WM53	Biolegend
176Yb	HLA-DQ	Tu169	Biolegend
209Bi	CD16 (FcgrIII)	3G8	Fluidigm

Supplementary Figure 1

A. DNA labeling

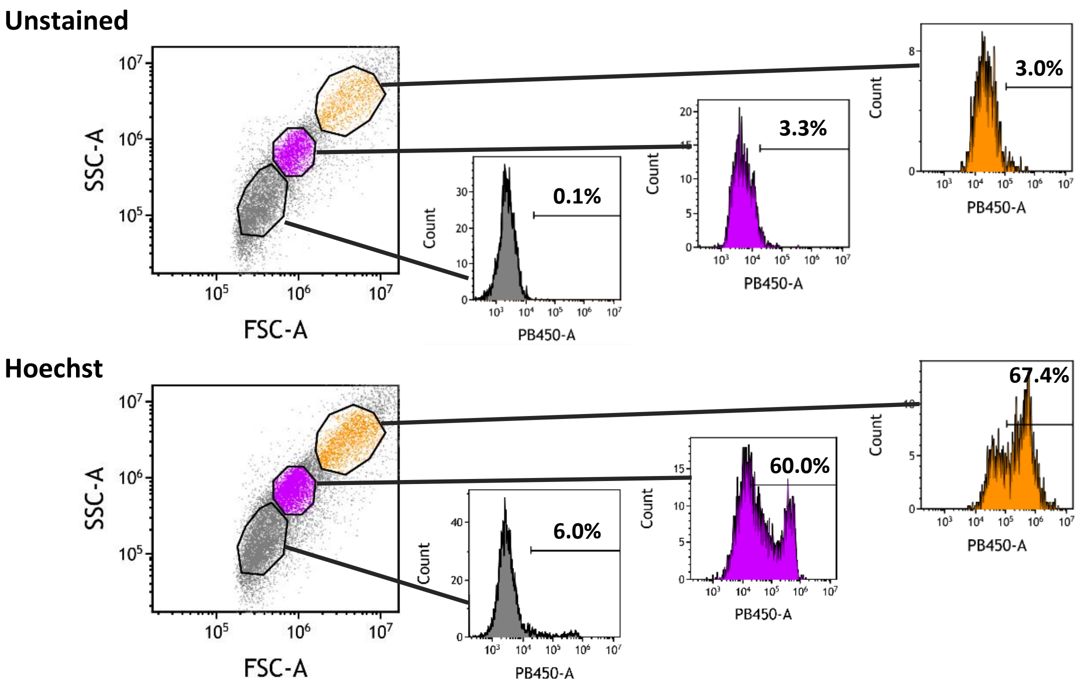

B. Blocking experiments

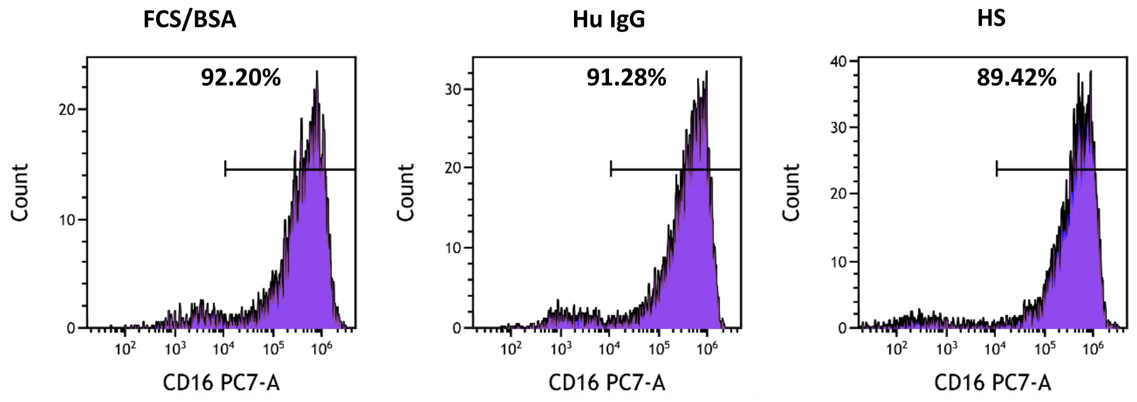

Supplementary Figure 1. Flow cytometry analysis of urine cells. **A. Flow cytometry gating strategy and DNA labeling**. Doublets were excluded in the FSC_{height}/FSC_{area} plots. Hoechst staining was analysed within three different gates in FCS/SSC as depicted. Upper panels correspond to unstained samples and lower panels to samples stained with 1/100 Hoechst 33342 dye. The percentage of Hoechst positive cells is shown for each histogram; the position of the marker was maintained in each region. The grey region corresponds to DNA-negative events. **B. Blocking experiments**. Different conditions for blocking of Fc receptors were tried: PBA [PBS supplemented with 0.5% bovine serum albumin (BSA), 1% FBS and 0.1% sodium azide], 10 mg/ml of purified human IgG and 10% human serum (HS). Cells were stained with antibodies against CD14 and CD16. The figure represents histograms of the CD16 staining within the CD14 positive cells. All the blocking conditions have similar staining pattern.

Mass cytometry data normalization

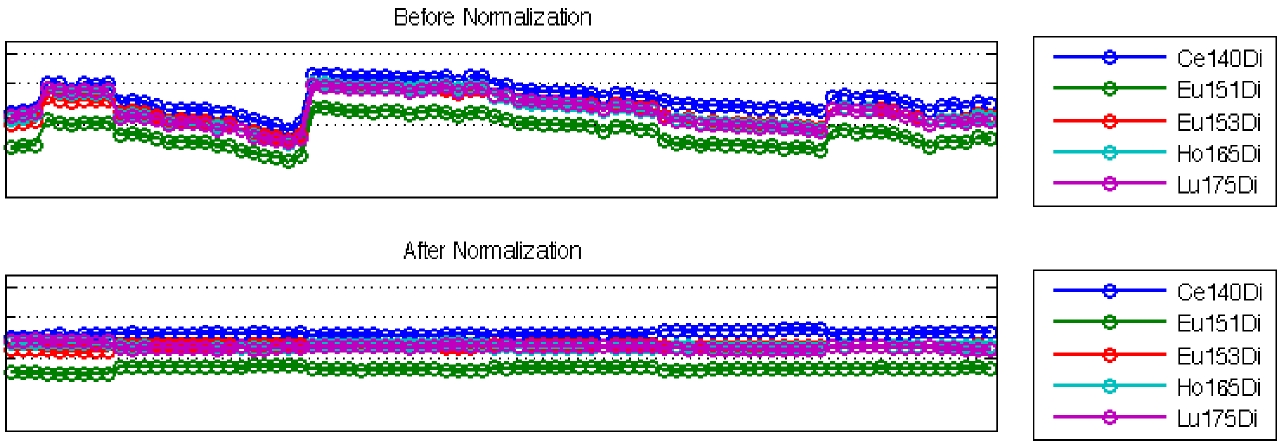

Supplementary Figure 2. Mass cytometry data normalization. Data collected in all CyTOF experiments were run through Normaliser v0.3.

Supplementary Figure 3

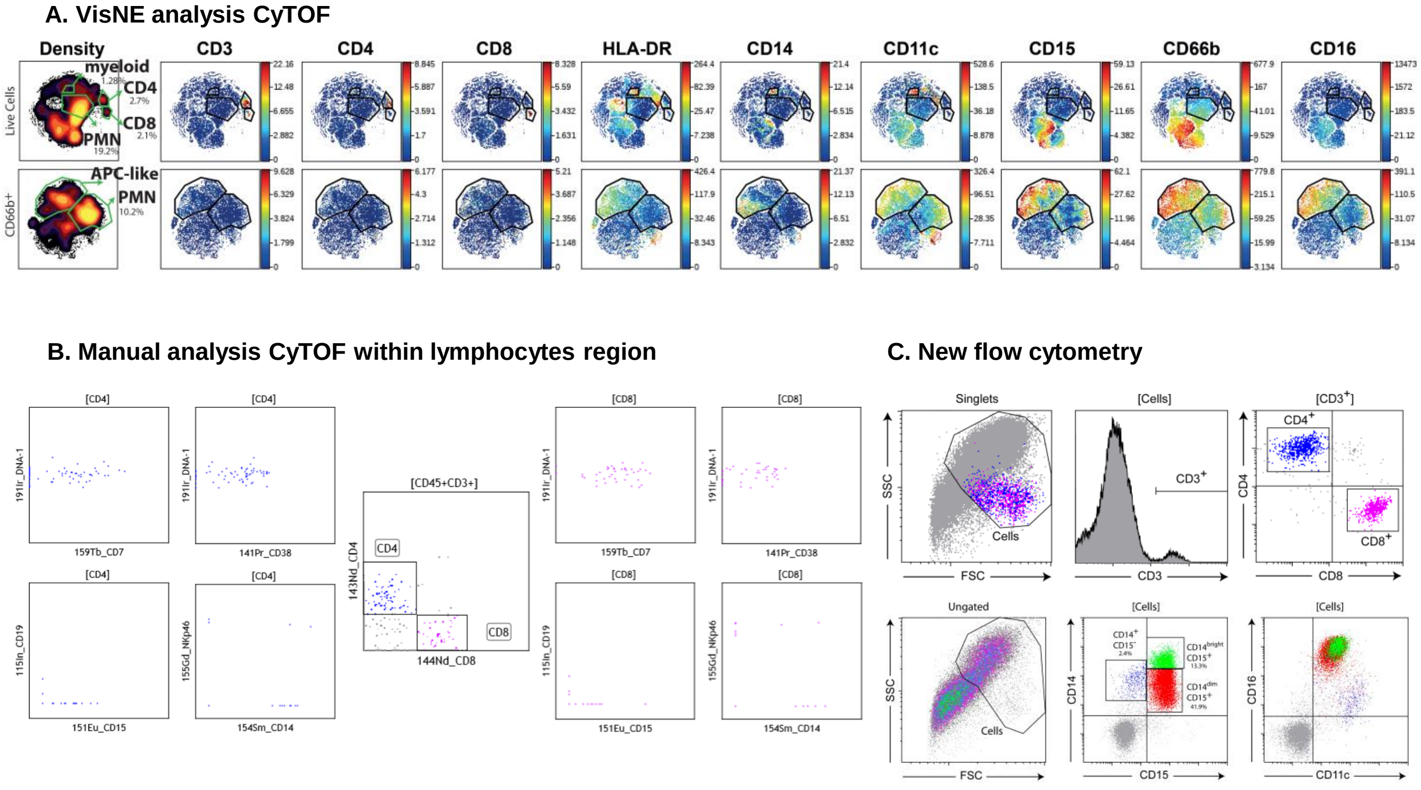

Supplementary Figure 3. Leukocytes released to urine analysed by CyTOF and flow cytometry. A. viSNE analysis. $CD3^+$ cells could be visualised in viSNE analyses and these lymphocytes could be classified as CD4 or CD8. Similarly, CD66b cells, also positive for CD15, were identified. **B. Manual analysis of CyTOF data.** CyTOF data were analysed using flow cytometry software to identify CD3 positive cells, $CD4^+$ and $CD8^+$. **C. Flow cytometry.** Cells from the same patient were analysed by flow cytometry to confirm the presence of CD3 lymphocytes. A population of $CD15^+CD14^+CD16^+$ cells was confirmed.

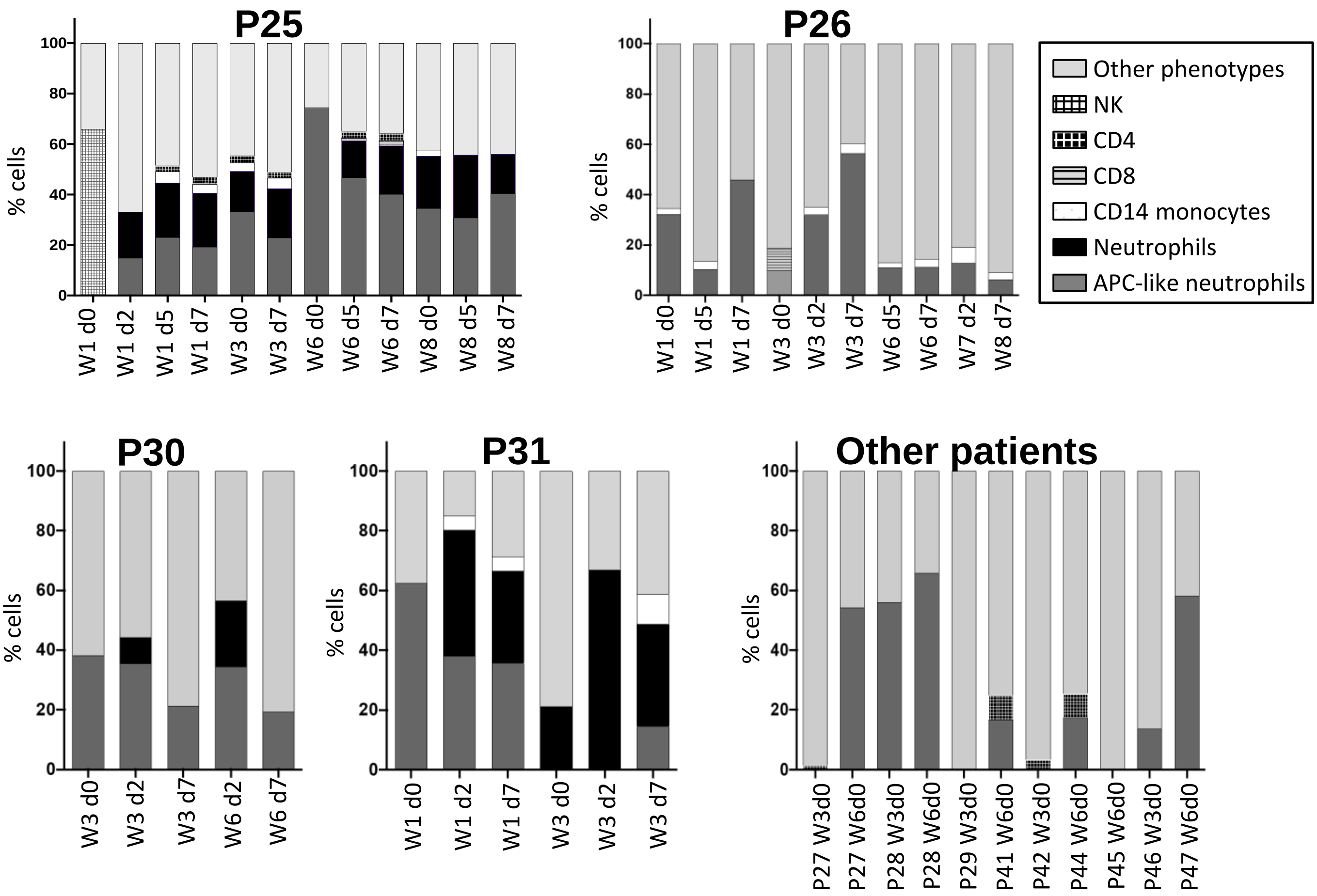

Supplementary Figure 4. Immune populations in different patients. Graphs represent the number of cell populations found in each patient in viSNE analysis of data obtained in CyTOF.
